# Oxytocin protective effects on zebrafish larvae models of autism-like spectrum disorder

**DOI:** 10.22038/IJBMS.2023.68165.14889

**Published:** 2023-03

**Authors:** Hooman Rahmati-Holasoo, Armin Salek Maghsoudi, Milad Akbarzade, Mahdi Gholami, Amir Shadboorestan, Faezeh Vakhshiteh, Maryam Armandeh, Shokoufeh Hassani

**Affiliations:** 1 Department of Aquatic Animal Health, Faculty of Veterinary Medicine, University of Tehran, Tehran, Iran; 2 Center of Excellence for Warm Water Fish Health and Disease, Shahid Chamran University of Ahvaz, Ahvaz, Iran; 3 Department of Toxicology and Pharmacology, Faculty of Pharmacy, Tehran University of Medical Sciences (TUMS), Tehran, Iran; 4 Department of Toxicology, Faculty of Medical Sciences, Tarbiat Modares University, Tehran, Iran; 5 Oncopathology Research Center, Iran University of Medical Sciences (IUMS), Tehran, Iran; 6 Toxicology and Diseases Group (TDG), Pharmaceutical Sciences Research Center (PSRC), The Institute of Pharmaceutical Sciences (TIPS), Tehran University of Medical Sciences (TUMS), Tehran, Iran; # These authors contributed eqully to this work

**Keywords:** Autism spectrum disorder, Oxytocin receptor, Shank3, Social behavior, Zebrafish

## Abstract

**Objective(s):**

Autism is a complicated neurodevelopmental disorder characterized by social interaction deficiencies, hyperactivity, anxiety, communication disorders, and a limited range of interests. The zebrafish (*Danio rerio*) is a social vertebrate used as a biomedical research model to understand social behavior mechanisms.

**Materials and Methods:**

After spawning, the eggs were exposed to sodium valproate for 48 hr, after which the eggs were divided into eight groups. Except for the positive and control groups, there were six treatment groups based on oxytocin concentration (25, 50, and 100 μM) and time point (24 and 48 hr). Treatment was performed on days 6 and 7, examined by labeling oxytocin with fluorescein-5-isothiocyanate (FITC) and imaging with confocal microscopy and the expression levels of potential genes associated with the qPCR technique. Behavioral studies, including light-dark background preference test, shoaling behavior, mirror test, and social preference, were performed on 10, 11, 12, and 13 days post fertilization (dpf), respectively.

**Results:**

The results showed that the most significant effect of oxytocin was at the concentration of 50 μM and the time point of 48 hr. Increased expression of *shank3a*, *shank3b*, and *oxytocin receptor* genes was also significant at this oxytocin concentration. Light-dark background preference results showed that oxytocin in the concentration of 50 µM significantly increased the number of crosses between dark and light areas compared with valproic acid (positive group). Also, oxytocin showed an increase in the frequency and time of contact between the two larvae. We showed a decrease in the distance in the larval group and an increase in time spent at a distance of one centimeter from the mirror.

**Conclusion:**

Our findings showed that the increased gene expression of *shank3a*, *shank3b*, and *oxytocin receptors *improved autistic behavior. Based on this study some indications showed that oxytocin administration in the larval stage could significantly improve the autism-like spectrum.

## Introduction

Autism spectrum disorder (ASD) is a neurodevelopmental brain disorder recognized by impaired social communication and interaction, anxiety, behavioral disorders, and obsessive interests causing reduced life quality (1, 2). Because of the reported increase in ASD prevalence, the disorder has attracted much attention and has become a top priority for many scientists (3). Although it is well acknowledged that ASD has a variety of causes, both genetic and environmental origins, a thorough understanding of the processes that drive atypical neurodevelopment is missing, and this may be due to the challenges in finding appropriate animal models and the neurobiological complexity in brain function (4, 5). There is a large body of evidence that ASD is a genetically-based disorder with a heritability of about 90% and is thought to be the most heritable brain disorder in humans affecting approximately 1–2% of the general population (6-9). Genetic investigations indicated that single-gene mutations cause changes in the developmental pathways of neuronal and axonal structures that are crucial in synaptogenesis (10, 11). ASD is most likely caused by interactions between several genes and variations in gene expression driven by epigenetic changes and environmental stimuli such as maternal age, nutrient deficiencies, medications, and exposure to toxicants(12). Despite recent advances in understanding the neurological foundations of ASD, there are currently no practical therapeutic approaches that target the core symptoms of ASD, including social communication issues and restricted/repetitive behavior (13). However, pharmacological therapies may be employed to alleviate related comorbidities but not core defects (14-16). Oxytocin is a neuropeptide mainly generated in the hypothalamus and released into the circulation via the posterior pituitary. Although oxytocin was formerly thought to have reproductive functions, new research reveals it is essential for social cognition and behavior. Oxytocin may enhance socially reinforced learning, benevolence, emotional empathy, and self-referential understanding, helping to promote positive social behaviors (17). These newly discovered oxytocin functions have led scientists to hypothesize about its therapeutic benefits for treating mental diseases with social behavior symptoms, such as social interaction disabilities and impaired communication skills observed in people with ASD (17-21). Previous clinical studies have described the efficacy of oxytocin in treating autistic symptoms (22-24). In those with ASD, oxytocin boosts the saliency and hedonic appraisal of social cues (25, 26). Nonetheless, further research on the effectiveness and mechanism of action is needed before oxytocin is approved for medical usage. Furthermore, there is mounting evidence that oxytocin regulates social bonding, affiliative motivation, and social recognition in animals by affecting its receptor (27-29). So, more research employing animal models is beneficial to speed up novel therapeutic development (30-34). In this regard, emerging experimental models utilizing zebrafish (*Danio rerio*) are rapidly gaining favor in neuroscience research to complement classic rodent models of brain disorders (35, 36). The importance of larval and adult zebrafish models in neuroscience has significantly increased because of their high degree of physiological and genetic similarity to humans and matching central nervous system (CNS) morphological structure (36, 37). Fast growth, high fertility, translucent embryos, and similar behavioral traits to rodents are the significant advantages of the zebrafish model (36, 38-40). Also, zebrafish are preferred over rats because of the high number of offspring utilized in scientific investigations and the ability to perform the assay repeatedly. As a result, zebrafish is gaining popularity as a potential model for ASD investigation (36, 41-43). One of the zebrafish toxicological models of ASD is exposure to neurotoxicant agents like sodium valproate (VPA), clinically applied as an epilepsy treatment and mood stabilizer. VPA exposure in the early stages of life leads to reduced hatching rates, lower motor function, and impaired social interaction without enhancing aggressiveness as adults (44, 45). This is a well-accepted method for the induction of autism-like models because it results in the behavioral and morphological abnormalities related to autism’s pathogenesis (1, 46, 47). VPA alters the expression of the ASD-related gene *shank3* and changes the frequency of *shank3* isoforms, perhaps affecting zebrafish embryonic development. Accordingly, both larvae and adult zebrafish lacking Morpholino shank3 have less social engagement, spend rarely around conspecifics, and exhibit repetitive swimming patterns (48). This study aimed to assess oxytocin delivery to the brain of zebrafish larvae by the drug labeling method and the effect of oxytocin treatment on behavioral parameters including light-dark background preference test, shoaling behavior, mirror test, and social preference. Also, the altered expression of *shank3a*, *shank3b*, and *oxytocin receptor* genes in the VPA-induced autistic zebrafish model have been evaluated. 

## Materials and Methods

***Chemicals***

Sodium valproate (VPA), oxytocin, fluorescein-5-isothiocyanate (FITC), phosphate buffer saline (PBS), DMSO, and all other chemicals used in this investigation were acquired from Sigma-Aldrich (GmbH, Munich, Germany). The primers were purchased from Pishagam Biotech Co. (Iran). iScript cDNA synthesis kit, TRIzol reagent, and 2X RT Pre-Mix were obtained from Biofact (China). Other reagents and chemicals were of analytical grade. The Research Ethics Committees of The Institute of Pharmaceutical Sciences, Tehran University of Medical Sciences (code number: IR. TUMS.TIPS.REC.1399.077) approved the work.

***Animals***

Adult wild-type zebrafish were kept and raised using conventional techniques, at a density of 1.5 fish per liter and a consistent light/dark cycle (14 hr:10 hr) (49). For reproducing, females and males (1:2) were placed in breeding tanks overnight and separated from fertilized eggs by a transparent barrier set the following day when the lights were switched on. After 15 min, fertilized eggs were harvested and placed in sterilized 6-well cell culture plates (20 fertilized eggs per well) and were kept at 28 °C with a regulated (14 hr:10 hr) light/dark cycle in incubators. Fertilized eggs were incubated in Biochemical Oxygen Demand (BOD) incubators at a density of 7 ml per larva until age 7 days post fertilization (dpf) (50). After that, they were immediately moved to a new tank with a larval density of one per 60 ml. The embryo’s daily visual evaluation under a dissection scope by an inverted stereomicroscope was conducted to assess general morphology and survival.

***Pharmacological induction***

VPA was given to chosen embryos from 0 to 48 hr post fertilization (hpf) at a concentration of 48 µM diluted in water based on a prior study (45). 

***Treatment***

**Figure 1:**
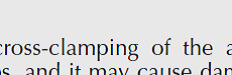
An overview of the experimental design and endpoints measured in zebrafish larvae exposed to sodium valproate and oxytocin during the experimental period

According to previous studies, oxytocin is active at room temperature in water for 21 days with normal function (51). Oxytocin was dissolved in water at concentrations of 100, 50, and 25 µM and then vortexed to achieve total dissolution. Embryos were divided into a positive control group (receiving sodium valproate), a control group, and six treatment groups with oxytocin (25, 50, and 100 µM with two exposure times of 24 and 48 hr). In groups of 24 hr, larvae are exposed to oxytocin on day 6 after fertilization, and in groups of 48 hr, larvae are exposed to oxytocin on 6 and 7 dpf. In addition, Figure 1 depicts the timeline for the experimental design. Due to the permeability of the blood-brain barrier up to 10 dpf in zebrafish larvae (52), oxytocin dissolved in water can penetrate directly into the brain tissue and induce possible therapeutic effects. Permeability was investigated through labeled oxytocin and imaging under confocal microscopy.

***Labeling***

FITC (10 mg) was dissolved in DMSO without water immediately before use. The FITC was then added to a ratio of 40–80 micrograms per milligram of oxytocin, and immediately after this step, the tube was wrapped in foil and finally stirred at room temperature for 1 hr. Unreacted FITC was separated and replaced in the mixture with filtration or dialysis gel in a 500 mM carbonate solution (pH 9.5).

***Confocal microscope***

Finally, the larvae were exposed to FITC-conjugated oxytocin on day 6, a 5-micrometer incision was made and photographed with a Nikon, Eclipse-Ti confocal microscope (Nikon, Japan) at 495 nm excitation and 519 nm emission.

***Larval social behaviors***

The larval zebrafish social behaviors apparatus we employed was smaller and more efficient than the adult phase zebrafish social behavior tools previously used in the literature (45). 

These analyses were conducted based on a previous study (53) with some modifications. We evaluated four high-output larval behaviors. EthoVision software version 11 (Noldus Information Technology, Wageningen, Netherlands) was used to analyze the larval movement and behavioral tests. 

*Light/dark background preference behavior*

Following the exposure period, incubation of the larvae in fish water (electrical conductivity up to 800 μS/cm) in a petri dish (50 ml, 40 larvae) until age 10 dpf for the light/dark background preference analysis was conducted between 11:00 and 13:30 Iran Standard Time (IRST). The device was a 24-well plate with half of each well colored black on the sides and bottom (outer surfaces) to provide balanced light and dark zones. The larvae entered each of the wells one by one, with 2 ml of fish water added to each well. The number of times the test larvae moved between light and dark areas and the number of larvae in the light area was recorded by motion tracking software (data collection every 60 seconds for 6 min). The average percentage of time spent in the light area and the average number of times passing through each treatment group’s light/dark area, which includes 24 larvae and three times repetitions, was examined (53).

*Shoaling behavior*

Ten larvae in each 9 cm diameter glass petri dish containing 25 ml of fish water were employed for shoaling test between 11 and 13:30 IRST at age 11 dpf. The parameters of the shoaling test were evaluated by inter-individual distance (IID) and nearest neighbor distance (NND) indices among a group of zebrafish. For each group, we monitored the movement of 10 larvae for 8 min, collecting data every 15 sec. The first 2 min were used to make adjustments in time division, and the remaining 6 min were used for analysis. Two trials were performed for each group; then, the experiments were combined (including 20 larvae in each group) and analyzed (53). The experiments were repeated three times for verification.

*Mirror attack behavior*

A mirror attack test was conducted utilizing a plate containing six wells constructed of ground glass to prevent fish from seeing each other. At age 12 dpf a one-way mirror was installed inside each well (11.2 (l) x 9 (w) x 2 (h) cm). Single larvae were moved to wells with 10 ml of fish water. Data were collected every 60 sec by carefully recording larval movements for 6 min. The subjects studied comprised attack (biting and lunges) in the mirror front and the time spent at a distance of less than or equal to 1 cm of the mirror (53). Data analysis was repeated for each treatment group (24 larvae) with three independent assays.

*Social contact*

A 6-well plate with 5 ml of fish water and two larvae in each well was used to evaluate social contact behavior at age 13 dpf. The movement of larvae was monitored for 6 min, and data were recorded every 60 sec. Social contact is a connection in which the distance between two larvae is less than or equal to the length of the body of one of the larvae; the interval of contact is essential (53). According to the study procedure, each treatment group consisted of 24 larvae, and three independent replications were recorded for each assay.

***qPCR analysis***

Expression levels of *shank3a*, *shank3b*, and *oxytocin receptor*s were measured by quantitative PCR (qPCR) using Takara SYBR Premix Ex Taq II (Takara, Japan) and the light cycler 96 apparatus (Roche, USA). Herein, 50 whole larvae from each group were obtained. Total RNAs were isolated using a Trizol reagent and the cDNA was prepared with a cDNA synthesis kit according to the manufacturer’s protocol. Primers are listed in Table 1. Relative mRNA expression levels were normalized to *β-actin* (*ACTB*) as a housekeeping gene by using the ΔΔCt method.

***Statistically analysis***

The data were expressed as mean ± standard deviation (SD). A one-way analysis of variance (ANOVA) was utilized for multiple comparisons, followed by Tukey’s test. *P*<0.05 was regarded as statically significant.

## Results

***Drug delivery assessment***

**Figure 2:**
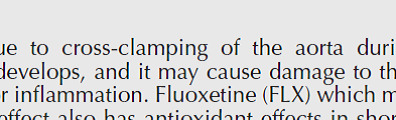
Confocal microscope images of larval brains taken at 20x and 40x magnifications in control groups (A and B) and FITC-conjugated oxytocin treatments (C and D). Green spots are the areas that oxytocin has reached

Based on the results of confocal microscopy imaging, we concluded that oxytocin reached the brain at the lowest concentration (25 μM) and the lowest exposure time (24 hr) in the compared control group. As unconjugated FITCs were isolated before exposure, all the colors seen in the brain are oxytocin conjugated to FITC (inset of Figure 2C, D).

***Behavioral assessment***

*Light/dark background preference behavior*

**Figure 3:**
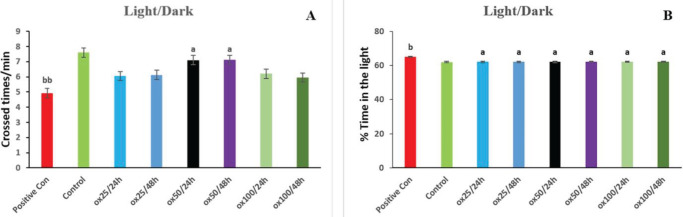
Protective effect of oxytocin in improving social behavior indices in terms of crossed times/min (A) and % of time spent in the light (B) on the Light/Dark background preference behavior. The values are displayed as mean ± SD (n=3). (a) *P*<0.05, compared with the positive control group. (b) *P*<0.05 and (bb) *P*<0.01 compared with control

According to the results depicted in Figure 3A, in the positive control group, the number of crossings between dark and light areas per minute has decreased significantly compared with the control (*P*<0.01), secondly in the group oxytocin 50/24 hr and oxytocin 50/48 hr the number of passes between the dark and light areas increased significantly compared with the positive control group (*P*<0.05). There was no statistically significant difference between the other treatment groups and the positive control group. Furthermore, as shown in Figure 3B, the time spent in the light area increased significantly in the positive control group compared with the control (*P*<0.05). When compared with the positive control group, all treated groups showed a significant decrement in the percentage of this parameter (*P*<0.05).

*Shoaling behavior*

**Figure 4:**
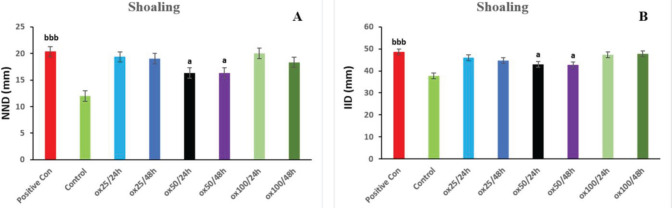
Protective effect of oxytocin in improving social behavior indices in terms of NND (A) and IID (B) on shoaling behavior. The values are displayed as mean ± SD (n=3). (a) *P*<0.05, compared with the positive control group. (bbb) *P*<0.001 compared with control

According to the results obtained in Figure 4A, in the positive control group, the NND parameter compared with the control has increased noticeably (*P*<0.001). Oxytocin 50/24 hr and oxytocin 50/48h groups significantly decreased the NND parameter compared with the positive control group (*P*<0.05). Furthermore, there was no substantial shift in the other treatment groups compared with the positive control group. According to the data shown in Figure 4B, the IID parameter increased considerably in the positive control group compared with the control (*P*<0.001). The IID index was significantly lower in the oxytocin 50/24 hr and oxytocin 50/48 hr groups compared with the positive control group (*P*<0.05). Compared with the positive control group, there was no dramatic change in the other treatment groups. 

*Mirror attack behavior*

**Figure 5:**
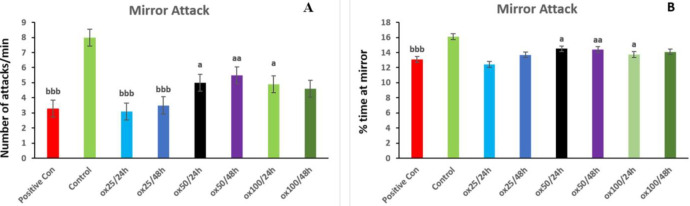
Protective effect of oxytocin in improving social behavior indices in terms of the number of attacks (A) and % of time at mirror (B) on Mirror attack behavior. The values are displayed as mean ± SD (n=3). (a) *P*<0.05, (aa) *P*<0.01 compared with the positive control group, and (bbb) *P*<0.001 compared with control

This behavioral test evaluated the number of attacks in the mirror and the percentage of time in the mirror area (≤ 1 cm away from the mirror). According to the results obtained in Figure 5A in the positive control group, the number of mirror attacks per minute has decreased significantly compared with the control (*P*<0.001). In oxytocin 50/24 hr, oxytocin 50/48 hr, and oxytocin 100/24 hr groups the number of mirror attacks per minute increased compared with the positive control group (*P*<0.05, *P*<0.01, and *P*<0.05, respectively). The other treatment groups did not significantly differ from the positive control group. The percentage of time spent near the mirror, which is considered to be less than or equal to 1 cm as a standard, decreased significantly in the positive control group compared with the control group (*P*<0.001) (Figure 5B).

In the oxytocin 50/24 hr, oxytocin 50/48 hr, and oxytocin 100/24h groups the time spent percentage in the area near the mirror (less than equal to 1 cm away from the mirror) increased significantly compared with the positive control group (*P*<0.05, *P*<0.01, and *P*<0.05, respectively). There was no significant difference in the other treatment groups compared with the positive control group.

*Social contact*

**Figure 6:**
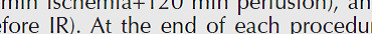
Protective effect of oxytocin in improving social behavior indices in terms of number of contacts/min (A) and % of time in contact (B) on Social contacts test. Values are displayed as mean ± SD (n=3). (a) *P*<0.05, (aa) *P*<0.01 compared with the positive control group, and (bbb) *P*<0.001 compared with control

According to Figure 6A, the number of contacts between two larvae in the positive control group dropped sharply compared with the control group (*P*<0.001). In the oxytocin 50/24 hr group and oxytocin 50/48 hr group, the number of contacts compared with the positive control group increased significantly (*P*<0.05 and *P*<0.01, respectively).

According to the results obtained in Figure 6B, the contact time of two larvae in the positive control group was significantly reduced compared with the control (*P*<0.001). The number of contacts in the oxytocin 50/24 hr group increased significantly compared with the positive control group (*P*<0.01). In the oxytocin 50/48 hr group, a significant increase in contacts compared with the positive control group was observed (*P*<0.05).

***qPCR analysis***

*Shank3a*

**Figure 7:**
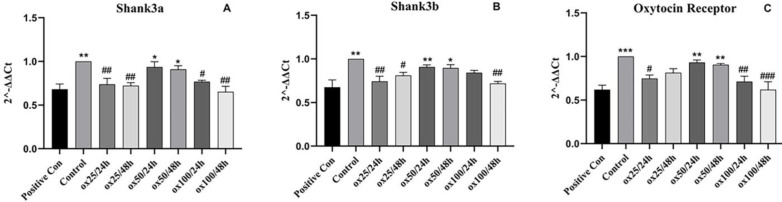
Effect of oxytocin exposure on the expression of genes involved in autism-like behavior in zebrafish larvae. shank3a (A), shank3b (B), and oxytocin receptor (C). The values are provided as mean ± SD (n=3). *P<0.05, **P<0.01, and ***P<0.001 compared with positive control group. #*P*<0.05, ##*P*<0.01, and ###*P*<0.001 compared with control, respectively

The expression of the *shank3a* gene in the positive control group (receiving sodium valproate) was significantly reduced compared with the control (not receiving sodium valproate) (*P*<0.01) (Figure 7A). Among the different doses of oxytocin, the dose of 50 µM in both exposure times (24 and 48 hr) was able to significantly increase the expression of the *shank3a* gene in comparison with the positive control group (*P*<0.05). Also, exposure to oxytocin at doses of 25 and 100 µM significantly reduced *shank3a *gene expression compared with the control. Exposure for 48 hr shows a more significant reduction, especially at the dose of 100 µM.

*Shank3b*

As shown in Figure 7B, the expression of the *shank3b* gene in the positive control group (receiving sodium valproate) was significantly reduced compared with the control (*P*<0.01). Among the different doses of oxytocin, a dose of 50 µM significantly increased the expression of the *shank3b* gene compared with the positive control group. This increase was more effective in 24-hr exposure (*P*<0.01) than in 48-hr exposure (*P*<0.05). Doses of 25 and 100 µM did not show such an effect. Also, exposure to oxytocin at doses of 25 and 100 µM significantly reduced *shank3a* gene expression compared with the control.

*Oxytocin receptor*

The expression of the *oxytocin receptor* gene in the positive control group (receiving sodium valproate) was significantly reduced compared with the control (*P*<0.001). Among the different doses of oxytocin, the dose of 50 µM was able to substantially increase the expression of the *oxytocin receptor* gene in comparison with the positive control group in both exposure times (24 and 48 hr, *P*<0.01) (Figure 7C). Also, exposure to oxytocin at 25 and 100 µM doses significantly reduced the *oxytocin receptor* gene expression compared with the control, which was more pronounced at the 100 µM dose.

**Table 1:** Primers sequence used in quantitative polymerase chain reaction (qPCR) experiments to study the interacting effects of sodium valproate and oxytocin on the expression of genes involved in the manifestation of autism spectrum disorders

**Primers**	**Sequence**
Zf *ACTB* Forward (accession number: NM_131031)	GAT CTT CAC TCC CCT TGT TCA
Zf *ACTB* Reverse	ATG TCT GGG TCG TCC AAC AA
Zf *shank3a* Forward (accession number: XM_017352009)	ACA GCG AAC TTC ACATCT GCT
Zf *shank3a* Reverse	GGC TGG GAA GGC TCA TGT TT
Zf *shank3b* Forward (accession number: XM_017355260)	CAA CCA GGA AGG CTG TGC TA
Zf *shank3b* Reverse	CCG GGA CGA ATA AGA AGG GG
Zf *oxytocin *Forward (accession number: XM_005166001)	TTC TCC GTG CAG ATG TGG TC
Zf *oxytocin *Reverse	CAA CCA GGA AGG CTG TGC TA

NND: Nearest Neighbor Distance; IID: Inter-Individual Distance

## Discussion

The main symptoms of ASD behavior are deficiency in social interactions, attention deficit hyperactivity disorder (ADHD), anxiety, mimicry, and repetitive behaviors (54). Valproate administration in embryonic cells, embryos, and rodents has developed animal models with similar behavioral effects (45). In the present study, zebrafish larvae were exposed to a concentration of 48 µM VPA which induced impaired social behavior. The findings of our study indicate that embryonic exposure to VPA results in behavior and physical characteristics similar to autism. In the preferred light/dark background preference test, zebrafish larvae exposed to VPA, showed a significant reduction in transition between light/dark areas and an increase in the percentage of residence time in the light area. There is an increase in movement between the light and dark areas in other animal models and no significant difference in the percentage of time spent in the light region (55). Possible reasons include differences in methodologies among laboratories (56, 57), differences between researchers (58), and adaptive ecological response. In shoaling assays, larvae exposed to VPA significantly increased NND and IID. The shoaling behavior of the individual group of fish indicated poor sociality, which was compatible with ASD characteristics. In the mirror attack experiment, larvae exposed to VPA had considerably fewer attacks on the mirror and a lower percentage of time spent in the mirror area, which was comparable with prior rodents (59) and zebrafish (43, 60) investigations of the autistic model. In measuring social contact, the frequency and duration of contact were significantly reduced as the decrease in the number of social behavior and a rise in the latency of social activities were observed in prenatal rats exposed to VPA (1). Oxytocin signaling disruption was not found to affect social preferences, such as shoaling behavior, in Ribeiro *et al*. (61) study, in contrast with our findings showing that oxytocin contributes to social preference in zebrafish larval behavior. Clearly, the treated groups (24 hr/50 µM) and (48 hr/50 µM) displayed decreased IID and NND parameters. A light-dark transition test in rodents is considered equivalent to a light/dark background preference test in fish, which was used by another study to investigate the effect of oxytocin on the autistic Chd8 haploinsufficiency mouse (62). ASD-like behavioral manifestations such as altered social behavior and anxiety were investigated in the mentioned study. A crucial finding in this study contradicted our results: when Chd8^+/∆SL^ male mice were treated with oxytocin intraperitoneally, they stayed longer in the light area, suggesting that oxytocin rescued anxiety-like behavior in male mice. According to the current study, VPA administration for ASD induction increased the percentage of time spent in the light area. Nevertheless, the percentage of time spent in the light area was significantly reduced by administration of oxytocin. The inconsistency between the results of rodent and zebrafish ASD models could be due to developmental differences, differences in methodologies employed by different laboratories, as well as ecological adaptations. For capturing prey and avoiding predators, diurnal species such as zebrafish rely on vision and well-lit environments (53). The mirror test, which includes the number of attacks on the mirror and approaches to the mirror and the percentage of time spent approaching, was used to study the function of oxytocin in treated larvae in the present study. The results clearly indicated that in zebrafish larvae that were only exposed to VPA, and ASD was induced in them, the mirror-biting frequency to mirror was significantly reduced. However, the number of attacks on the mirror increased significantly among the three groups treated with oxytocin. One of the studies that were conducted in the field of investigating the induction of enhanced aggressive behavior in zebrafish through exposure to tributyltin revealed interesting results in the mirror test. Researchers found that using low and high concentrations of tributyltin significantly increased zebrafish presence in the mirror area and the number of mirror attacks (63).

 The oxytocinergic pathway is involved in a wide range of complicated social interactions and behaviors (64). Oxytocin is a neuropeptide that plays a role in regulating multiple facets of social behavior. Apart from its critical role as a hormone, oxytocin has an important function in the CNS as a neurotransmitter peptide. Most neurons end up in the posterior pituitary gland; others are found in the CNS (65). Endogenous central oxytocin plays a significant role in developing social behavior from a physiological point of view (66-68). According to evidence, the oxytocinergic system may be implicated in the pathophysiology of certain neuropsychiatric disorders such as schizophrenia and autism (69). No definitive treatment pathway has been found so far, despite multiple studies conducted to find a standard therapeutic pathway for social behavior disorders. The current investigation is noteworthy because the effects of oxytocin as an oxytocin receptor agonist on social behavior in a novel animal model have been scrutinized. The results of our study revealed the positive effect of oxytocin treatment (50 μM) against the negative effects of exposure to VPA as a model of ASD. The oxytocin treatment model regulated the time elapsed in the area closest to the matched group and the time spent in the area closest to the mirror image. Furthermore, we found that oxytocin, as an oxytocin receptor agonist, improved social and aggressive behavior. The effect of oxytocin receptors on behavior and social aggression has only been studied pharmacologically in a few previous studies. The results of this study were in agreement with the finding of another study (70), which examined the mechanisms underlying the evolution and maintenance of shoaling behavior and social preferences in zebrafish aged between two to eight weeks by examining the oxytocin receptors *oxtrl* and *oxtr*. An experiment in which the oxytocin receptor was knocked out led to increased group spacing and decreased polarization 8 weeks post-fertilization in a shoal of 20 fish (70). Oxytocin receptors play an important role in regulating social behavior, as demonstrated by this study. These effects are not purely prosocial or antisocial but are linked to both the fish’s age as well as their social context. In 2014, Mooney *et al.* (71) demonstrated that oxytocin enhances social interaction, which can be inhibited by co-administering an oxytocin antagonist. Likewise, in another study, the oxytocin peripheral antagonist administration was shown to reduce the time elapsed for convergence between zebrafish species (72). According to a study carried out in 2017, there is a statistically significant relationship between oxytocin levels and social disorders in children (73). Some studies have shown that irregular processing and retention of social information in people with Asperger’s disorder or autism is managed by administering a therapeutic dose of oxytocin (26, 74, 75). Similar procedures have been used in behavioral studies to demonstrate that oxytocin administration can improve social abilities in zebrafish and rodents (75-77). Previous studies showed that oxytocin increased social preference in zebrafish, and its antagonists in a dose-dependent manner, inhibited the effect induced by neuropeptides (13, 78, 79). In line with the above studies, our study showed that oxytocin in specific concentrations and time intervals reduced autistic zebrafish larvae’s impaired social communication and aggressive behavior. Several studies indicate that mutations in *SH3 *and multiple ankyrin repeat domains 3 (*shank3*) were important in autism and ASD (44, 80-82). The orthologous of the human *shank3* gene is duplicated in zebrafish as *shank3a *and* shank3b* (83). Different mutations have been detected, such as the whole gene and two frameshift deletions in the *shank3 *gene (84).  Among the different doses of oxytocin, the dose of 50 µM in both exposure times (24 and 48 hr) was able to significantly increase the expression of the *shank3a* and *shank3b* genes in comparison with the positive control group. This study showed that a 50 µM dose of oxytocin could modulate the decrease in the expression of the above genes altered due to exposure to VPA, but not observed at 25 and 100 µM doses. Unlike mammals with only one OT receptor gene, zebrafish have two orthologous receptor genes (oxtr, oxtrl). The zebrafish and mammalian oxytocin have different potency. The potency (EC50) of zebrafish oxytocin is about 3 nM in both oxtr and oxtrl. However, this potency for mammalian oxytocin is ~ 48 and 35 nM for oxtr and oxtrl, respectively (81). On the other hand, the effect of oxytocin on *Shank3a/b* gene expression was not dose-dependent. These results were consistent with the results of behavioral studies. In this way, social behaviors improve by increasing the expression of the above genes. In 2021, Lee *et al*. (85) manipulated the expression of *shank3*-related genes in the ASD model of adult male mice and concluded that a disorder in the *shank3* pathway led to a decrease in neurons encoding experience and interaction with other mice. In parallel, the encoding level of neurons related to individual experience increased. Mutations in the* shank3* gene, which is responsible for encoding an important postsynaptic protein in glutaminergic synapses, assist in understanding the genetic basis of ASDs. In this regard, a study demonstrated that social memory was significantly impaired in mice whose *shank3* isoforms were destroyed by removing spanning exons 11-21 of *shank3* (86). Additionally, learning disabilities and the development of anxiety-like behavior were observed (87). It was also previously reported that a mutation in a single copy of *shank3* could occur on chromosome 22q13, leading to language and social contact disorders associated with the development of ASD (88). Considering that Phelan-McDermid syndrome (PMS) is caused by a mutation in the *shank3* gene and is one of the most common reasons for ASD and mental retardation, Harony-Nicolas *et al*. designed an examination in which the effect of oxytocin on the PMS rat model was assessed. In rats treated with intracerebroventricular (ICV) oxytocin, long-term social recognition memory and social attention process exhibited significant improvement (89). In addition to oxytocin, several factors affect how the oxytocinergic system treats ASD. One of the most important factors is the level of oxytocin receptors and their expression. The defect in *oxytocin receptor* expression and deficit of this receptor directly disrupts the signaling pathway of this neuropeptide. Since the oxytocin receptor contains several single-nucleotide polymorphisms (SNPs), researchers have genotypically linked the SNPs to various behaviors such as social contact; therefore, different aspects of social behavior are associated with the level of oxytocin receptors (90).

## Conclusion

The findings of this study suggest that oxytocin may have a better permeability of the blood-brain barrier (BBB) during the fetal period and exert its effects in this way, leading to improved behavioral symptoms and modulation of changes due to decreased expression of *shank3a, shank3b, and oxytocin receptor*. In general, this study showed that starting treatment with oxytocin after induction of autism-like in zebrafish with VPA in the early days of zebrafish larval life can significantly improve behavioral symptoms and expression of genes involved in autism-like behavior. From a futuristic perspective, it is suggested that to compare the effect of oxytocin and evaluate the impact of age, the autism-like model is developed in both groups of zebrafish during embryonic and adult zebrafish, and then the effect of oxytocin is examined.

## Authors’ Contributions

HRH supervised; ASM prepared and visualized the draft and editing of the manuscript; MA designed the experiments; MG performed experiments and collected data; AS and FV analyzed and interpreted the results; MA edited the manuscript, SH investigation, conceptualization, methodology, project administration, and funding acquisition.

## Funding

This research was in part supported by a grant from The Institute of Pharmaceutical Sciences (TIPS), Tehran University of Medical Sciences, Iran (coded 99-2-263-49063), received by Shokoufeh Hassani.

## Conflicts of Interest

The authors declare that they have no conflicts of interest in this work.
